# Expression and prognostic value of the transcription factors EGR1 and EGR3 in gliomas

**DOI:** 10.1038/s41598-020-66236-x

**Published:** 2020-06-09

**Authors:** Arnon Møldrup Knudsen, Ida Eilertsen, Susanne Kielland, Mikkel Warming Pedersen, Mia Dahl Sørensen, Rikke Hedegaard Dahlrot, Henning Bünsow Boldt, Sune Munthe, Frantz Rom Poulsen, Bjarne Winther Kristensen

**Affiliations:** 1 Department of Clinical Research, University of Southern Denmark, Odense, Denmark; 2 Department of Pathology, Odense University Hospital, Odense, Denmark; 3 Department of Oncology, Odense University Hospital, Odense, Denmark; 4 Department of Neurosurgery, Odense University Hospital, Odense, Denmark

**Keywords:** Cancer, Biomarkers, Medical research

## Abstract

Most glioblastoma patients have a dismal prognosis, although some survive several years. However, only few biomarkers are available to predict the disease course. EGR1 and EGR3 have been linked to glioblastoma stemness and tumour progression, and this study aimed to investigate their spatial expression and prognostic value in gliomas. Overall 207 gliomas including 190 glioblastomas were EGR1/EGR3 immunostained and quantified. A cohort of 21 glioblastomas with high P53 expression and available tissue from core and periphery was stained with double-immunofluorescence (P53-EGR1 and P53-EGR3) and quantified.EGR1 expression increased with WHO-grade, and declined by 18.9% in the tumour periphery vs. core (P = 0.01), while EGR3 expression increased by 13.8% in the periphery vs. core (P = 0.04). In patients with high EGR1 expression, 83% had methylated MGMT-promoters, while all patients with low EGR1 expression had un-methylated MGMT-promoters. High EGR3 expression in MGMT-methylated patients was associated with poor survival (HR = 1.98; 95%CI 1.22–3.22; P = 0.006), while EGR1 high/EGR3 high, was associated with poor survival vs. EGR1 high/EGR3 low (HR = 2.11; 95%CI 1.25–3.56; P = 0.005). EGR1 did not show prognostic value, but could be involved in MGMT-methylation. Importantly, EGR3 may be implicated in cell migration, while its expression levels seem to be prognostic in MGMT-methylated patients.

## Introduction

Gliomas are the most common primary brain tumours, with the WHO grade IV glioblastoma multiforme (GBM) being the most malignant. In GBMs the current standard treatment with radical surgical resection, radiation and temozolomide therapy results in a median survival of approximately 15 months^1–3^, although some patients survive several years after diagnosis. Only few prognostic biomarkers are of use in daily practise, like the methylation status of O-6-Methylguanine-DNA Methyltransferase (MGMT)^4^ and mutational status of the Isocitrate dehydrogenase 1/2 genes^5^. Identification of additional novel biomarkers is therefore crucial in order to better stratify the patients.

GBMs are characterized as highly vascularized, heterogeneous tumours with a profoundly infiltrative nature, leading to accelerated and aggressive disease progression. Nearly all GBMs recur after initial treatment efforts due to migrating tumour cells, which infiltrate the adjacent healthy brain parenchyma and escape surgical excision as well as radiation and temozolomide therapy. We have previously shown that these migrating tumour cells have a stem-cell like phenotype and are highly tumourigenic *in vivo*^6^.

Early growth response protein 1 (EGR1) and Early growth response protein 3 (EGR3) are C2H2-type zinc-finger proteins, which belong to the EGR-protein family of transcription factors. EGR1 has been proposed to regulate the expression of genes involved in cell proliferation, growth and cell differentiation^7,8^. In addition, EGR3 has been implicated in immune regulation^9–11^ and cell migration^12,13^. Recently, EGR1 has been linked to the proliferation and self-renewal of brain tumour initiating cells^14,15^, suggesting that early growth response proteins may be implicated in the maintenance of niche-populations of GBM cells. Based on this, we hypothesized that EGR1 and EGR3 may be associated with tumour progression, possibly mediated through promotion of tumour cell migration, and thus hold prognostic value in gliomas. EGR1 expression levels have been associated with patient survival in gastric^16^, colorectal^17,18^, and ovarian cancer^19^, while EGR3 levels have been shown as a prognostic marker in gastric cancer^20^ and breast carcinomas^21^.

Since GBM cells diffusely migrate into the surrounding brain, and a fraction of these migrating tumour cells survive current treatment modalities, this population of treatment resistant tumour cells most likely plays a major role in tumour progression and ultimately patient prognosis. Investigations of novel targets or biomarkers rarely consider this aspect of heterogeneity. This is a major limitation in some studies, since the poor understanding of the biology of migrating tumour cells may help these cells escape novel therapies.

The objectives of this study were I) to investigate the protein expression of EGR1 and EGR3 in gliomas, II) to compare protein expression levels in tumour cells located in central, intermediate and peripheral tumour areas to uncover potential differential expression patterns in migrating and non-migrating tumour cells, and III) to investigate the prognostic potential of EGR1 and EGR3, including their combined prognostic value.

## Materials and methods

### Patient inclusion

Archived formaline-fixed paraffin embedded glioma tissue samples from all consecutive patients that underwent brain tumour surgery at the Department of Neurosurgery, Odense University Hospital, in The Region of Southern Denmark, Denmark, between January 1, 2010 and December 31, 2014 were examined. A total of 207 patients had sufficient amounts of resected tumour tissue to be included in this study. All patients were followed from the date of surgery until death or date of censoring (March 1, 2018). A total of 34/207 patients (16.4%) were still alive at date of censoring. Median follow up was 15.0 months (range: 0.1–96.7 months). None of the patients had a record of previous brain malignancies, and no treatment was given prior to surgery. Only GBM patients were included in the multivariate cox-regression, and of these 144/190 patients (75.8%) received the standard of care Stupp treatment regimen^2^, 23/190 patients (12.1%) received 34 Gy radiation, 5/190 (2.6%) received 59 Gy radiation, while 18/190 (9.5%) received no treatment and only had a biopsy specimen taken. All included tumour samples were re-classified according to the 2016 WHO classification of tumours of the central nervous system^22^. Patient characteristics are presented in Table 1. All experimental procedures in this study, including use of patient material and data, have been performed according to local and national guidelines and regulations, covered by permissions from the Danish Data Inspection Authority (approval number 16/11065) and the Regional Scientific Ethical Committee of the Region of Southern Denmark (approval number S-20150148). All tissue specimens used in this study were obtained after informed consent, as part of the standard of care therapy.

**Table 1:** Patient characteristics.

Variable	Number (%)	Male/Female	Dead/alive	Age, mean	Median survival in months	EGR1%, mean	EGR3%, mean
All patients	207 (100)	119/88	173/34	62.51	15.11	26.59	51.0
**WHO grade**							
II	12 (5.8)	8/4	2/10	40.78	54.24	7.12	37.5
III	5 (2.4)	3/2	3/2	54.68	15.24	9.85	51.0
IV	190 (91.8)	108/82	168/22	64.09	14.49	28.26	51.66
**MGMT status (WHO IV)**							
Methylated	94 (49.5)	51/43	76/18	63.08	18.14	38.41	51.0
Un-methylated	71 (37.4)	46/25	70/1	64.95	13.99	15.72	52.0
Unknown	25 (13.1)	11/14	22/3	65.45	11.20	25.71	50.0
**ECOG Performance status (WHO IV)**							
0–1	135 (71.1)	82/53	115/20	62.49	16.89	29.49	52.0
2–4	54 (28.4)	25/29	52/2	68.65	7.59	25.38	51.0
Unknown	1 (0.5)	1/0	1/0	34.92	45.60	17.61	20.0
**Post-surgical treatment**							
Yes	172 (90.5)	99/73	151/21	63.96	14.98	28.18	52.0
Stupp	144 (75.8)	87/57	123/21	61.89	16.03	28.44	53.0
Radiation	28 (14.7)	11/17	28/0	74.07	10.55	28.39	47.0
No	18 (9.5)	9/9	17/1	65.36	5.65	29.06	49.0

### Tissue preparation

Tissue microarrays (TMA) were made by sampling 2–3 cylindrical tissue cores with a diameter of approximately 5 mm from each patient tumour sample. A total of 183 patients had sufficient tissue for three tumour cores, while 24 patients had sufficient tissue for two tumour cores. The tumour cores were embedded into new paraffin TMA blocks and cut into histological sections of 3 µm on a microtome. The TMA tissue sections were then mounted on glass slides and stored at −80 °C until immunohistochemical staining.

### Immunohistochemistry

Tissue sections were subject to deparaffinisation and heat-induced epitope retrieval (HIER) with either CC1- (EGR1) or TEG15 (EGR3) buffers using a microwave oven. Endogenous peroxidase activity was blocked with peroxidase inhibitor. Incubation with primary EGR1 antibody 1:50 (clone: 15F7, Cell Signaling Technology) or EGR3 antibody 1:2000 (clone: PA5–40841, ThermoFisher Scientific) was done using the BenchMark ULTRA platform (Ventana Medical Systems) with the OptiView-DAB detection system for EGR1 stainings, and the AutostainerPlus platform (DAKO, Glostrup, Denmark) with the catalyzed signal amplification system II for EGR3 stainings. Colon tissue served as a positive external control and was mounted on each slide. A tissue multiblock containing 27 different normal tissues and 12 different cancers served as both negative and additional positive control. Controls were systematically included in every staining procedure. All stained slides were digitalized using the NanoZoomer 2.0HT digital image scanner (Hamamatsu, Japan).

### Automated quantitative image analysis of EGR1 stainings

The images were imported into the Visiopharm Integrator System Software 6.6.1 (Visiopharm, Hoersholm, Denmark), and each TMA core was manually evaluated to exclude necrotic areas and artefacts. A software-based cell classifier was programmed to identify the fraction of EGR1 positive cells and their mean staining intensity. To minimize the risk of detecting false positive cells, a threshold based on staining intensity was applied, thereby excluding all cells with staining intensity below this threshold. The performance of the classifier was assessed by manually examining all TMA cores after classification. A total of 29 TMA cores presented with insufficient classification, and therefore a slightly modified version of the classifier was applied on these cores for a more accurate result. Examples of tissue sections with the applied classifier are shown in Supplementary Fig. 1A–D.

### Semi-quantitative scoring of EGR3 stainings

The EGR3 stainings were heterogenous and some tumour cores presented with a distinct nuclear staining pattern, while others showed diffuse cytoplasmic staining. An adequate Visiopharm cell classifier could not be applied to these stainings, and therefore a semi-quantitative assessment was used; Each TMA core was scored with regard to three different categories: 1) Estimation of the fraction of EGR3 positive nuclei (5% intervals), 2) the mean EGR3 intensity of positive cells, and 3) the area fraction of diffuse cytoplasmic EGR3 staining. Each category was given a score from 0–3, and the TMAs were furthermore divided into 3 groups based on their total score: group 1 = 0–3 points, group 2 = 4–6 points, group 3 = 7–9 points. A supplementary analysis of the fraction of EGR3-positive nuclei was performed in order to further dichotomize groups, with the median value used as cut-off. The scoring system with examples of scored tumour cores is outlined in Supplementary Fig. 1E–G. The researcher scoring the stainings was blinded to patient diagnoses and outcome, and reproducibility of the scoring system was tested by independently scoring all TMA cores twice.

### Double immunofluorescence and automated quantitative image analysis of nuclear EGR1 and EGR3 protein expression in central vs. migrating tumour cells

The original cohort of 207 gliomas was screened for tumours with high expression levels of P53 protein (≥60% positive cells in central tumour areas) and simultaneous presence of areas with diffuse tumour infiltration. A subset of 21 GBMs fulfilled these criteria, and was included in the cohort. In these tumours, P53 expression was utilised to pin-point tumour cells in order to enable exact measurement of the tumour cell population through exclusion of P53-negative glial cells and neurons.

For P53/EGR1 double fluorescence stainings, deparaffinisation and HIER was performed, followed by blocking of endogenous peroxidase activity. Slides were then incubated for 60 min with primary ready-to-use P53 antibody (clone: DO7, Ventana Medical Systems), on the AutostainerPlus staining platform. Antibody detection was performed using the Catalyzed Signal Amplification II kit conjugated with FITC (CSA II, Dako). Following a second HIER, sections were incubated for 60 minutes with primary EGR1 antibody, 1:50, followed by detection with the anti-rabbit Tyramide Signal Amplification System Cyanine-5 (Perkin Elmer, USA). Nuclei were counterstained with VECTASHIELD Mounting Medium containing 4,6-diamidino-2-phenylindole (DAPI) (VWR International, USA).

For P53/EGR3 double-fluorescence stainings, deparaffinisation and HIER was performed as previously, and slides were incubated with primary P53 antibody for 32 minutes on the Ventana Discovery Ultra staining platform. Antibody detection was performed with DISCOVERY OmniMap anti-Ms HRP coupled with the DISCOVERY FAM Kit. After a second HIER, sections were incubated for 32 minutes with primary EGR3 antibody, 1:2000, and detection performed with DISCOVERY OmniMap anti-Rb HRP coupled with the DISCOVERY Cy5 Kit. Nuclei were counterstained as previously.

Bright field super images of all whole tissue sections were acquired at 1.25X magnification using the Visiopharm software coupled with a Leica DM6000 B microscope equipped with an Olympus DP72 camera and a Ludl motorized stage. Regions of interests (ROI) including central tumour area, areas with intermediate tumour cell infiltration and peripheral tumour, were manually outlined for each slide. The software was set to sample 10 images at 20X magnification from each ROI using a Meander number based sampling algorithm. All sampled images were reviewed to ensure acquisition of at least 5 acceptable images including a minimum of 20% tumour area from each ROI. Areas containing necrosis, vessels, bleeding, and artefacts were excluded from analysis. A cell-classifier was developed to distinguish between DAPI-stained nuclei, P53-positive nuclei, EGR1/3-positive nuclei and P53 + /EGR1/3+ double-positive nuclei (Supplementary Fig. 2). The area fractions and mean intensities of positive nuclei were quantified in all images.

### Statistical analyses

Statistical analyses were performed with GraphPad Prism 5.01 and STATA 15.0. Comparison of mRNA and protein expression levels was performed with One-way-Anova tests and Tukey´s Post-tests for data with Gaussian distribution and Kruskal-Wallis tests and Dunn´s multiple comparison tests for data with non-Gaussian distribution. EGR1 and EGR3 correlation was investigated by Spearman rank correlation. Assessment of overall patient survival was performed with Kaplan-Meier estimators and Log-rank tests with EGR1/EGR3 expression values dichotomised at the median. Cox proportional hazards regression was performed to investigate and identify independent prognostic variables. The two radiation dosage regimens were combined into one common variable in the regression model. All assumptions in the Cox-regression model were tested, and a significant interaction between age and MGMT-methylation status was found. This interaction is outlined in Supplementary Fig. 3A. Data from The Cancer Genome Atlas (TCGA) GBM dataset and Ivy Glioblastoma Atlas Project (Ivy GAP)^23^ was accessed through GlioVis^24^ and used to supplement and elucidate relevant findings. P-values < 0.05 were considered statistically significant. Error-bars represent mean ± SEM.

### Ethics

The study was approved by the Danish Data Inspection Authority (approval number 16/11065) and the Regional Scientific Ethical Committee of the Region of Southern Denmark (approval number S-20150148).

## Results

### EGR1 and EGR3 protein expression in gliomas

**Figure 1:**
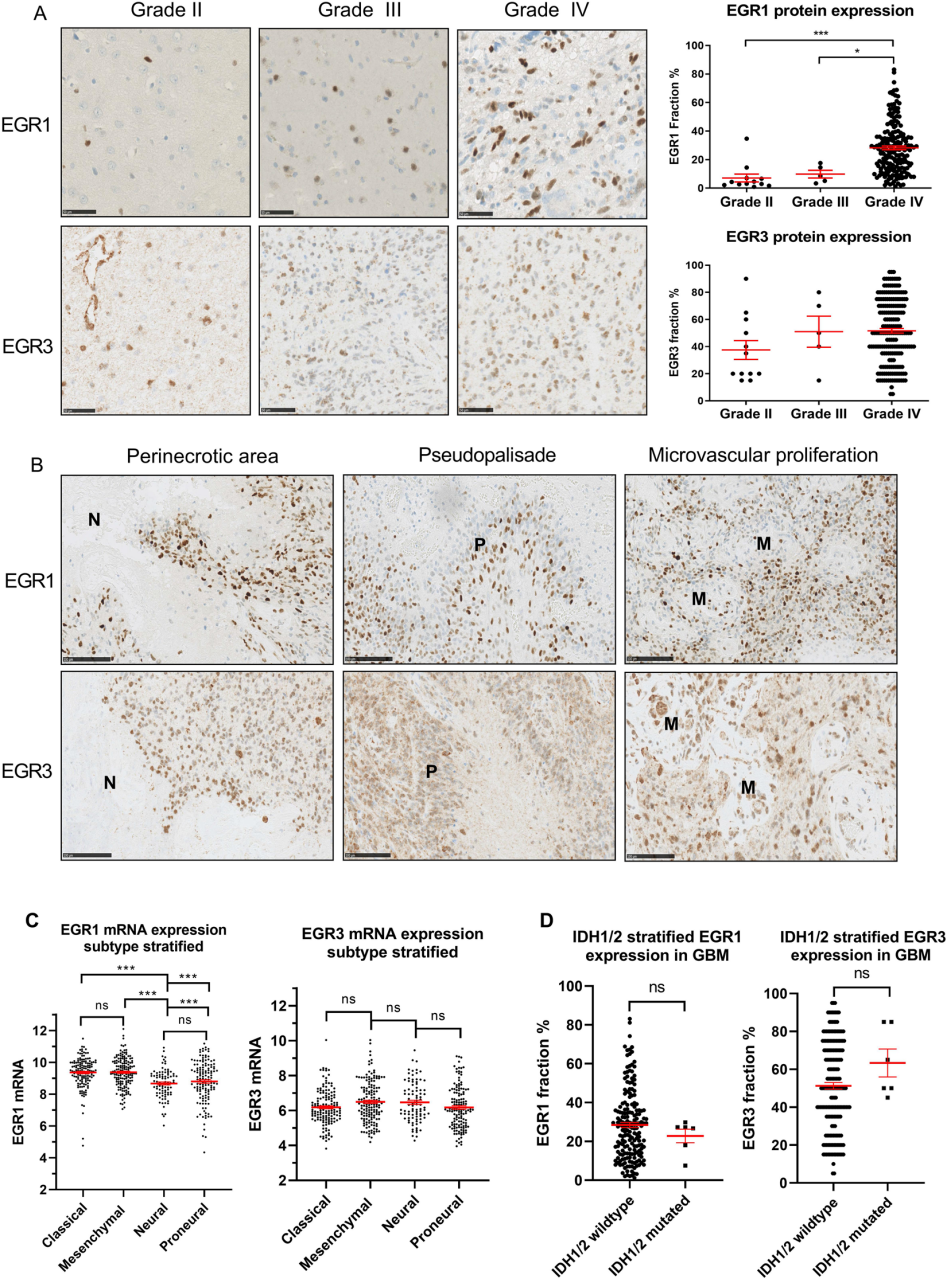
EGR1 and EGR3 protein expression in gliomas. **(A)** EGR1 and EGR3 expression levels displayed in representative grade II, III and IV gliomas. Images were acquired at 40X magnification with scale bars = 50 μm. EGR1 protein expression significantly increased with WHO grade and the highest expression levels were found in glioblastomas. EGR3 protein expression levels were independent of WHO grade. (**B)** Expression of both EGR1 and EGR3 was seen in areas with characteristic histological traits of GBMs, including areas with necrosis, pseudopalisades and around microvascular proliferations. Images were acquired at 30X magnification with scale bars = 100 μm. N = Necrosis. P = pseudo-palisade. M = microvascular proliferation. (**C)** Verhaak subtype stratification of EGR1 mRNA TCGA data showed that neural and proneural glioblastomas have slightly lower EGR1 mRNA levels compared to classical and mesenchymal tumors, while EGR3 was equally expressed in all subtypes. (**D)** Stratification of EGR1 and EGR3 protein levels based on IDH1/2 mutational status in the patient cohort. No significant changes in EGR1 or EGR3 protein levels were observed. * = P < 0.05. ** = P < 0.01. *** = P < 0.001.

EGR1 protein expression was found in all 207 gliomas, but with considerable inter-tumour variation (Fig. 1). The fraction of EGR1 positive cells ranged from 1–83% and significantly increased with WHO grade (P < 0.001) (means: grade II = 7.1%, grade III = 9.9%, grade IV = 28.3%, Fig. 1A). A significant difference between grade II and grade IV (P < 0.001) and grade III and grade IV (P < 0.05) was found, while grade II and grade III tumours did not differ significantly. The same results were found when analysing TCGA EGR1 mRNA data, which furthermore showed that the expression of EGR1 mRNA in normal brain tissue was significantly lower than in tumour tissue (data not shown). EGR3 protein expression was also found in all 207 gliomas with positive cell fractions varying from 5–95%. The mean variation between separate scorings of the EGR3-positive cell fractions was 7.3%. No significant association between EGR3 protein fraction and WHO grade was found (P = 0.13) (means: grade II = 37.5%, grade III = 51.0%, grade IV = 51.7%, Fig. 1A), and this was also the case for EGR3 mRNA data from TCGA (data not shown). In GBMs, expression of both proteins was found in peri-necrotic areas, pseudo-palisades and around local microvascular proliferations (Fig. 1B). No correlation between EGR1 and EGR3 protein expression levels was found (data not shown). Stratification of EGR1 and EGR3 mRNA data from TCGA by Verhaak subtypes^25^ showed that neural and proneural GBMs had slightly lower levels of EGR1 mRNA compared to classical and mesenchymal tumours, while EGR3 mRNA levels were equally expressed in all tumour subtypes (Fig. 1C). When stratifying GBMs based on mutational status of IDH1/2, no significant differences in EGR1 or EGR3 protein levels were found in IDH1/2 wildtype vs. IDH1/2 mutated tumours (Fig. 1D).

### **EGR1 and EGR3proteinexpression in central, intermediate, and peripheral tumour cells**

**Figure 2:**
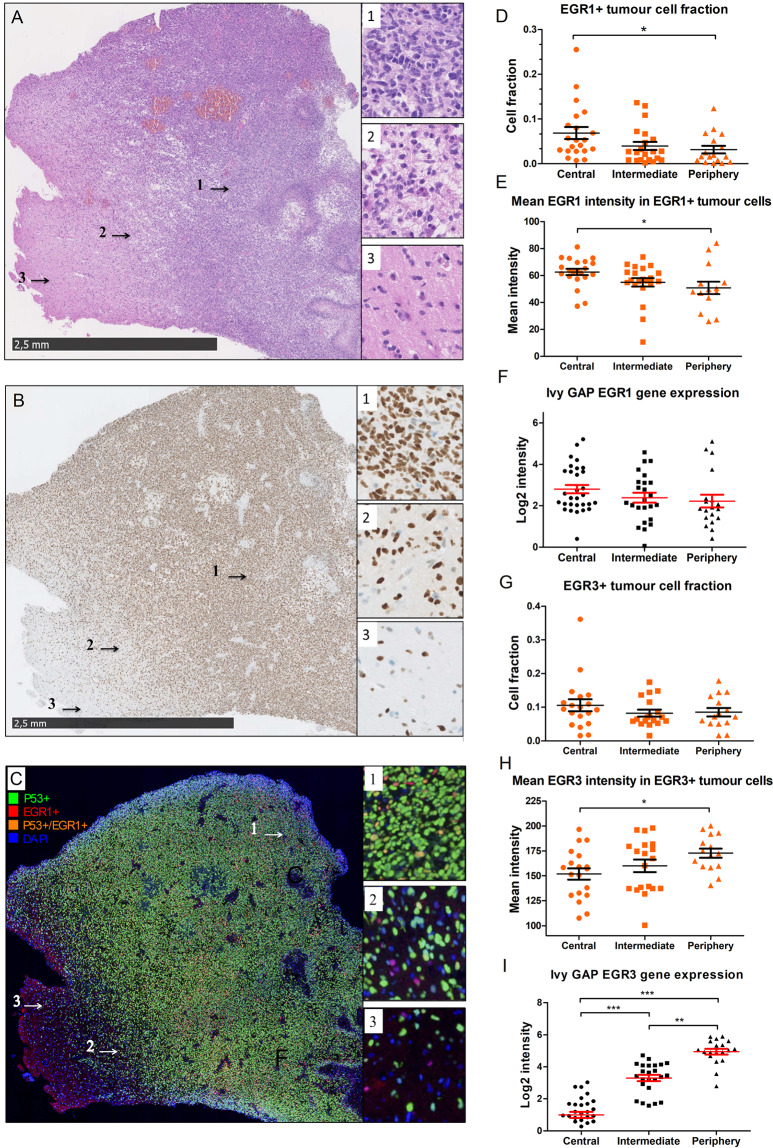
Expression of EGR1 and EGR3 in infiltrating tumour cells. **(A–C)** Representative HE, P53, and double-fluorescence images with P53/EGR1 on tissue sections from one of the tumours included in the cohort. 1 = central tumour area. 2 = intermediate tumour area. 3 = peripheral tumour area. Scale bar = 2.5 mm. DAPI-stained nuclei = blue, P53-positive nuclei = green, EGR1/3-positive nuclei = red, double-positive nuclei, i.e. positive for both P53 and EGR1/3 = orange. (**D,E)** The EGR1 fraction and staining intensity in EGR1 + tumour cells was significantly reduced from central tumour to periphery. (**F)** Ivy GAP data showed a trending but non-significant decline in EGR1 mRNA levels between central tumour and tumour periphery. (**G,H)** The EGR3 fraction remained constant across the different tumour regions, while the mean staining intensity increased significantly in peripheral tumour cells compared to central tumour cells. **I)** Ivy Gap EGR3 mRNA data also showed an increase in EGR3 intensity in the intermediate and peripheral areas compared to central tumour. * = P < 0.05. ** = P < 0.01. *** = P < 0.001.

As expected, the mean fraction of tumour cells, identified by P53, significantly declined in both the intermediate tumour area (P < 0.05) and in the tumour periphery (P < 0.001) compared to the central tumour areas (Fig. 2A–C). The mean fraction of EGR1 + tumour cells significantly declined from central tumour to periphery (5.3% vs. 1.6%, P < 0.05, Fig. 2D). When examining the mean EGR1 staining intensity in EGR1 + tumour cells, an intensity decline of 18.9% was found from central tumour (mean intensity = 62.6 AU) to periphery (mean intensity = 50.8 AU, Fig. 2E). Ivy GAP mRNA data showed a trending decline in EGR1 mRNA levels from central tumour to intermediate and periphery, however, the results were non-significant (P = 0.07, Fig. 2F). The mean fraction of EGR3 + tumour cells did not change significantly across the different tumour regions (central = 10.6%, intermediate = 8.2%, periphery = 8.5%, Fig. 2G). The mean EGR3 staining intensity in peripheral EGR3 + tumour cells (mean intensity = 172.7 AU) significantly increased by 13.8% when compared to central tumour cells (mean intensity = 151.8 AU, P = 0.04), indicating that peripheral tumour cells had higher expression of EGR3 protein compared to central tumour cells (Fig. 2H). Ivy GAP EGR3 mRNA data also showed a significant increase in mean EGR3 mRNA levels across the different tumour regions (central vs. intermediate P < 0.001; central vs. periphery P < 0.001; intermediate vs. periphery P < 0.01, Fig. 2I).

### **EGR1 and EGR3 expression and overall patient survival**

In grade II (n = 12) and grade III gliomas (n = 5), no association between patient survival and EGR1 protein fraction nor EGR1 staining intensity was found. Results for EGR3 nuclear fraction, both as 5% incremental values and by semi quantitative scores 1–3, as well as nuclear intensity score and cytoplasmic area fraction score were also non-significant (data not shown).

**Figure 3:**
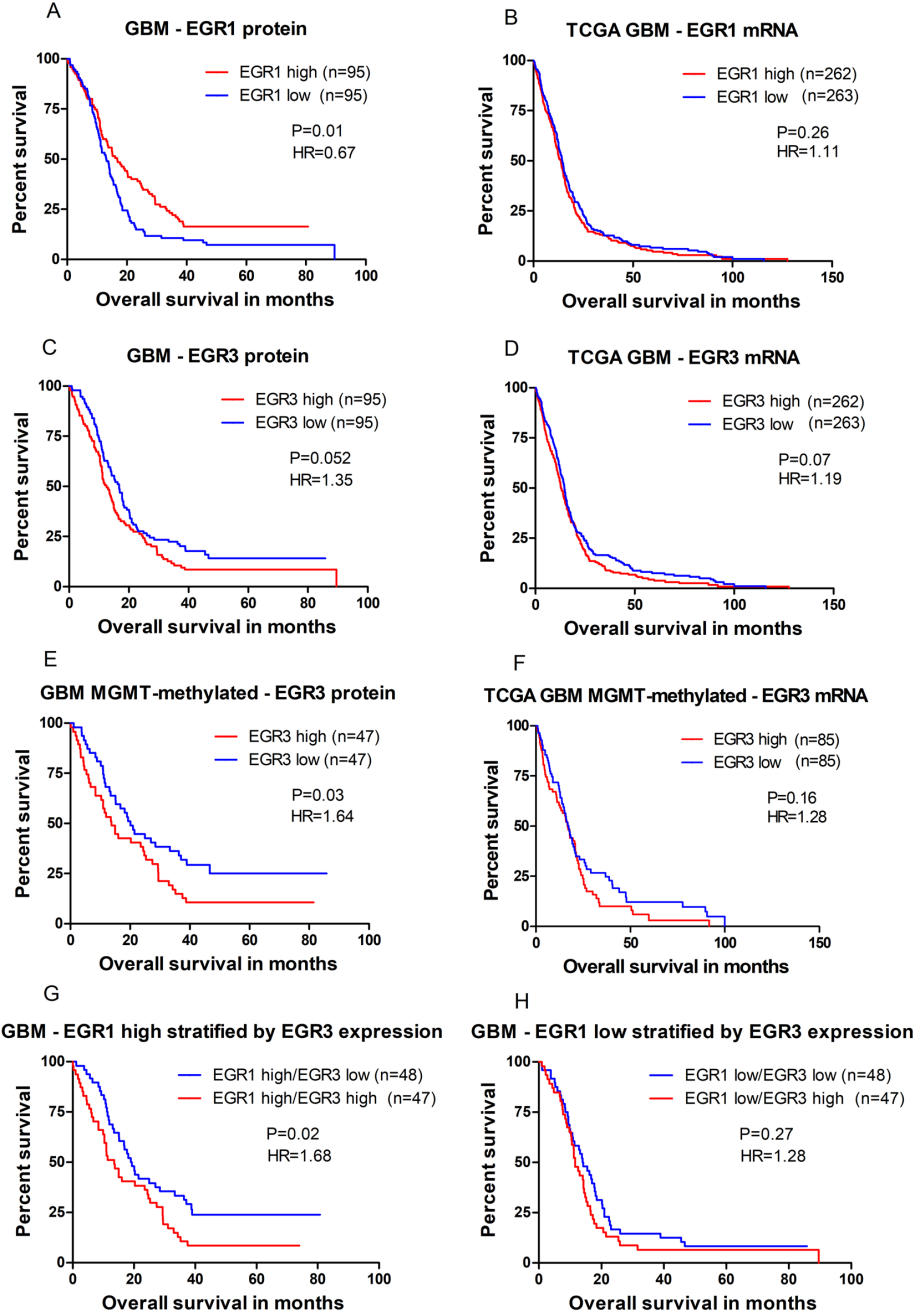
Overall patient survival in different subgroups. Patient survival illustrated for different EGR1 and EGR3 subgroups in glioblastoma patients both measured by positive cell fractions found in the immunostainings and by mRNA expression data from TCGA. All groups were dichotomized at the median. (**A)** GBM patients with a high EGR1 nuclear fraction showed a significantly better prognosis compared to patients with a low EGR1 fraction. (**B)** EGR1 mRNA levels from TCGA did not show any difference in survival between the two groups. (**C)** Patients with a high EGR3 nuclear fraction were borderline significant of having a poor prognosis compared to patients with a low EGR3 fraction. (**D)** EGR3 mRNA levels from TCGA showed the same trend as EGR3 protein levels. (**E)** MGMT-gene promoter methylated patients with a high EGR3 fraction lived significantly shorter than patients with a low EGR3 fraction. (**F)** The same trend was found in EGR3 mRNA data from TCGA, although results were non-significant**. G)** When sub-stratifying patients from the EGR1 high group by EGR3 expression, the group with high EGR3 expression had significantly shorter survival compared to patients with low EGR3 expression. H) Sub-stratification of patients from the EGR1 low group by EGR3 expression did not result in survival differences between the two groups. HR = Hazard ratio.

**Figure 4:**
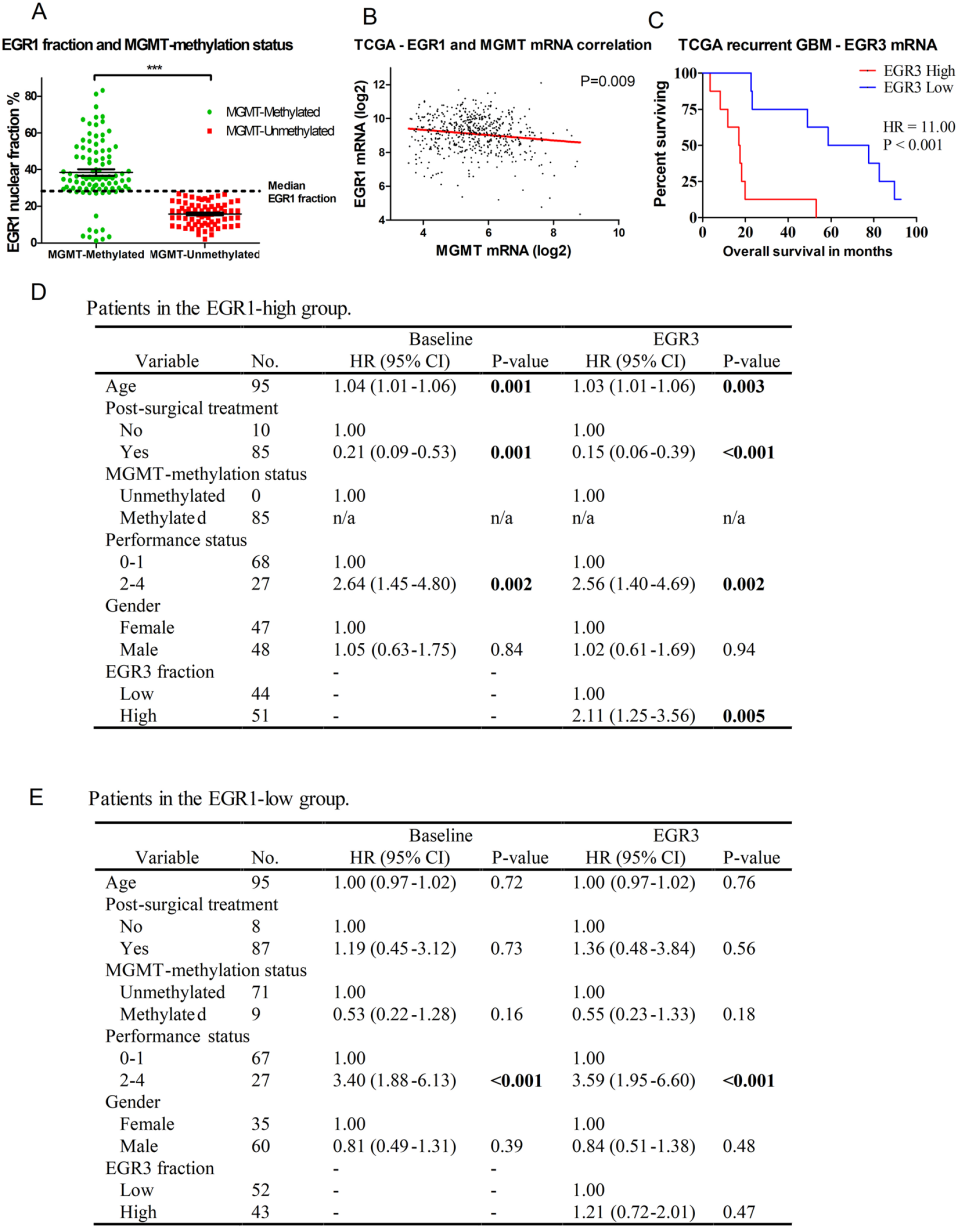
MGMT-methylation distribution and multivariate cox-regression for EGR1/EGR3 combinations. **(A)** The distribution of MGMT-methylation status was highly skewed when looking at the two EGR1 groups: The majority of patients in the EGR1 high group had a methylated MGMT-promoter, while 100% of patients in the EGR1 low group had un-methylated MGMT-promoters. (**B)** TCGA mRNA data showed a significant inverse correlation between EGR1 and MGMT mRNA levels. (**C)** TCGA data showed that patients with recurrent GBM and high EGR3 mRNA levels had significantly shorter survival compared to patients with low EGR3 mRNA levels. (**D,E)** Multivariate Cox-regression of the different EGR1 and EGR3 combinations showed that patients with EGR1 high/EGR3 high had a worse prognosis than patients with EGR1 high/EGR3 low. * = P < 0.05. ** = P < 0.01. *** = P < 0.001.

In GBMs (n = 190), a high EGR1 protein fraction was associated with longer overall survival when compared to a low EGR1 fraction: Median survival 16.76 vs. 13.11 months (HR = 0.67; 95%CI = 0.49–0.91; P = 0.01, Fig. 3A). However, when adjusting for known clinical parameters in multivariate cox-regression, the results were non-significant (P = 0.95, Table 2A), and EGR1 mRNA data from TCGA did not show any difference in overall survival between the two groups (Fig. 3B). When subdividing the tumours based on MGMT-methylation status, no difference in survival was found when looking at MGMT methylated (Table 2B) and un-methylated groups (Table 2C). Interestingly, 78/94 (83%) of patients with methylated MGMT-promoters were in the EGR1 high group with a mean EGR1-positive cell fraction of 38.4%, while 71/71 (100%) of patients with un-methylated MGMT-promoters were in the EGR1 low group with a mean EGR1 fraction of 15.72% (Fig. 4A). TCGA mRNA data showed a significant inverse correlation between EGR1 and MGMT mRNA levels (Fig. 4B).

**Table 2:** Multivariate Cox-regression for glioblastoma patients.

Variable	No.	Baseline		EGR1		EGR3	
		HR (95% CI)	P-value	HR (95% CI)	P-value	HR (95% CI)	P-value
**(A) All patients**							
Age	190	1.02 (1.01–1.04)	**0.004**	1.02 (1.01–1.04)	**0.004**	1.02 (1.01–1.04)	**0.003**
**Post-surgical treatment**							
None	18	1.00		1.00		1.00	
Stupp	144	0.51 (0.27–0.95)	**0.03**	0.51 (0.27–0.95)	**0.03**	0.54 (0.29–1.00)	**0.05**
Radiation	28	0.95 (0.45–1.97)	0.88	0.95 (0.45–1.98)	0.89	1.06 (0.50–2.27)	0.16
**MGMT-methylation status**							
Unmethylated	71	1.00		1.00		1.00	
Methylated	94	0.51 (0.36–0.74)	**0.001**	0.54 (0.24–1.20)	0.13	0.51 (0.35–0.74)	**0.001**
**IDH1/2 mutation**							
No	184	1.00		1.00		1.00	
Yes	6	0.13 (0.03–0.56)	**0.006**	0.13 (0.03–0.56)	**0.006**	0.14 (0.03–0.62)	**0.01**
**ECOG Performance status**							
0–1	135	1.00		1.00		1.00	
2–4	54	2.78 (1.81–4.31)	**<0.001**	2.79 (1.81–4.31)	**<0.001**	2.72 (1.76–4.21)	**<0.001**
**Gender**							
Female	82	1.00		1.00		1.00	
Male	108	0.94 (0.67–1.33)	0.27	0.94 (0.67–1.33)	0.27	0.94 (0.66–1.32)	0.70
**EGR1 fraction**							
Low	95	—	—	1.00		—	—
High	95	—	—	0.95 (0.43–2.11)	0.91	—	—
**EGR3 fraction**							
Low	95	—	—	—	—	1.00	
High	95	—	—	—	—	1.26 (0.90–1.77)	0.18
**(B) Patients with methylated MGMT-promoter**.							
Age	94	1.04 (1.01–1.06)	**0.001**	1.04 (1.01–1.06)	**0.001**	1.04 (1.01–1.06)	**0.001**
**Post-surgical treatment**							
None	8	1.00		1.00		1.00	
Stupp	76	0.20 (0.07–0.59)	**0.003**	0.20 (0.07–0.59)	**0.004**	0.12 (0.04–0.39)	**<0.001**
Radiation	10	0.42 (0.15–1.14)	0.09	0.42 (0.15–1.14)	0.09	0.35 (0.13–0.98)	**0.045**
**IDH1/2 mutation**							
No	92	1.00	n/a	1.00	n/a	1.00	n/a
Yes	2	n/a		n/a		n/a	
**ECOG Performance status**							
0–1	66	1.00		1.00		1.00	
2–4	28	2.71 (1.39–5.29)	**0.003**	2.71 (1.39–5.29)	**0.003**	2.22 (1.10–4.45)	**0.03**
**Gender**							
Female	43	1.00		1.00		1.00	
Male	51	1.07 (0.66–1.75)	0.77	1.07 (0.66–1.75)	0.77	0.99 (0.61–1.61)	0.96
EGR1 fraction		—	—			—	—
Low	9	—	—	1.00		—	—
High	85	—	—	0.99 (0.44–2.22)	0.99	—	—
EGR3 fraction		—	—	—	—		
Low	45	—	—	—	—	1.00	
High	49	—	—	—	—	1.97 (1.19–3.28)	**0.009**
**(C) Patients with un-methylated MGMT-promoter**.							
Age	71	0.99 (0.97–1.03)	0.94	0.99 (0.97–1.03)	0.94	1.00 (0.97–1.03)	0.96
Post-surgical treatment							
None	8	1.00		1.00		1.00	
Stupp	52	1.02 (0.42–2.47)	0.96	1.02 (0.42–2.47)	0.96	1.09 (0.42–2.85)	0.86
Radiation	11	2.08 (0.66–6.57)	0.21	2.08 (0.66–6.57)	0.21	2.24 (0.66–7.67)	0.19
**IDH1/2 mutation**							
No	67	1.00	**0.01**	1.00	**0.01**	1.00	**0.01**
Yes	4	0.11 (0.02–0.60)		0.11 (0.02–0.60)		0.11 (0.02–0.59)	
**ECOG Performance status**							
0–1	52	1.00		1.00		1.00	
2–4	19	3.43 (1.66–7.10)	**0.001**	3.43 (1.66–7.10)	**0.001**	3.53 (1.67–7.48)	**0.001**
**Gender**							
Female	25	1.00		1.00		1.00	
Male	46	0.95 (0.54–1.66)	0.84	0.95 (0.54–1.66)	0.84	0.97 (0.54–1.73)	0.92
EGR1 fraction		—	—			—	—
Low	71	—	—	1.00		—	—
High	0	—	—	n/a	n/a	—	—
EGR3 fraction		—	—	—	—		
Low	39	—	—	—	—	1.00	
High	32	—	—	—	—	1.10 (0.64–1.90)	0.72

**(A)** Cox-regression for all 190 included glioblastomas showed independent prognostic value of patient age, Stupp treatment regimen, MGMT-methylation status, IDH1/2 mutational status and ECOG performance status. A high EGR1 fraction was found non-significant. (**B)** Cox-regression including patients with a methylated MGMT-promoter. Independent prognostic value was found for patient age, Stupp treatment regimen, ECOG performance status and EGR3 fraction. (**C)** Cox-regression including patients with an un-methylated MGMT-promoter. Independent prognostic value was found for IDH1/2 mutational status and ECOG performance status. n/a = not applicable. HR = Hazard ratio. CI = Confidence interval. Significant p-values are marked with bold numbers. Survival data was adjusted for known clinical parameters including patient age, post-surgical treatment regimens, MGMT-methylation status, ECOG performance status, IDH1/2 mutation status, gender as well as EGR1 and EGR3 fractions. All Cox-regressions were first performed as a baseline-model and subsequently with addition of EGR1 or EGR3 respectively.

As opposed to results for EGR1, a high EGR3 protein fraction seemed to be associated with poor patient survival, although just short of being significant: Median survival 12.55 vs. 16.72 months (HR = 1.35; 95%CI = 0.99–1.84; P = 0.052, Fig. 3C). In multivariate analysis, results were non-significant (P = 0.11, Table 2). TCGA data showed the same trend of high EGR3 mRNA levels being associated with poor prognosis, but results were non-significant (P = 0.07, Fig. 3D). No association with patient survival was found for EGR3 nuclear fraction score, staining intensity scores, cytoplasmic staining scores, presence/absence of cytoplasmic staining or score groups based on total points achieved (Supplementary Fig. 3B–F). When subdividing the tumours based on MGMT-methylation status, the MGMT-methylated group with a high EGR3 cell fraction had a significantly shorter survival compared to the group with a low EGR3 cell fraction: Median survival 13.77 vs. 20.07 months (HR = 1.64; 95%CI = 1.04–2.58; P = 0.03, Fig. 3E). This result remained significant in multivariate cox-regression (P = 0.006, Table 2B). TCGA data showed a similar, but non-significant, trend for EGR3 mRNA levels in MGMT methylated GBM (P = 0.16, Fig. 3F). In the MGMT-unmethylated group, no survival difference was found between EGR3 high vs. EGR3 low expression levels: Median survival 13.27 vs. 13.21 months (HR = 1.09; 95%CI = 0.68–1.75; P = 0.72). Furthermore, high EGR3 mRNA levels were associated with a significantly shorter survival in recurrent GBMs (HR = 11.00, P < 0.001, Fig. 4C).

### Combined prognostic value of EGR1 and EGR3 in glioblastomas

The various combinations of EGR1 and EGR3 revealed that the groups differing the most were patients with high EGR1 levels, sub-stratified by their EGR3 expression (Fig. 3G). In the group with high EGR1 and simultaneous high EGR3 expression had significantly shorter survival compared to the group with high EGR1 and simultaneous low EGR3 expression: Median survival 13.54 vs. 19.14 months (HR = 1.68; 95%CI = 1.08–2.62; P = 0.02). In multivariate analysis, the difference remained significant (P = 0.005, Fig. 4D). In patients with low EGR1 expression, sub-stratification by EGR3 expression (Fig. 3H) was not significantly associated with any difference in overall survival: Median survival 14.00 vs. 11.60 months (HR = 1.28; 95%CI = 0.84–1.97; P = 0.27, Fig. 4E).

## Discussion

The expression of EGR1 in gliomas has previously been investigated by Sakakini *et al*.^14^ and Mittelbronn *et al*.^26^, who found EGR1 expression in 82% and 100% of tumours respectively, which is similar to our findings of expression in all 207 investigated tumours. Both studies found a positive correlation between EGR1 fraction and WHO grade, which is also in accordance with our results.

We found that EGR1 protein expression decreased in migrating tumour cells, and a possible explanation for this finding could be that cells in the peripheral tumour regions are less exposed to the active tumour microenvironment present in central tumour, and thereby less exposed to cytokine signalling and hypoxia, which are known inducers of EGR1 expression^27,28^.

The prognostic value of EGR1 in gliomas was also investigated by Mittelbronn *et al*.^26^ and Sakakini *et al*.^14^ who found that high EGR1 levels were associated with improved overall survival and progression free survival, respectively. In this study, we found a significant association between high EGR1 expression and improved overall patient survival in univariate analysis; however, the results were non-significant when adjusting for confounders. MGMT methylation status is known as a strong independent predictor of both progression-free survival and overall survival in GBM patients^29^. Nearly all patients with high EGR1 expression had a methylated MGMT promoter, while most patients with low EGR1 expression had an unmethylated MGMT promoter. This uneven distribution of MGMT methylation status explains why the EGR1 fraction had no prognostic value after adjustment for MGMT methylation status. Mittelbronn *et al*.^26^ and Sakakini *et al*.^14^ did not include MGMT-methylation status in their studies, which most likely explains the differences compared to our study. The finding of a very high fraction of MGMT-methylated tumours having simultaneous high EGR1 expression raises the question whether EGR1 is involved in methylation of the MGMT promoter. Data from TCGA showed a significant inverse correlation between EGR1 and MGMT mRNA levels, which supports this hypothesis, and warrants further investigation of the interaction between EGR1 and MGMT.

EGR1 has been reported to act as a tumour suppressor in several cancers^30–32^, however, in prostate cancer, EGR1 has been proposed to be an oncogene^33,34^, which may suggest a tissue specific biological function. In gliomas, it has previously been shown that overexpression of EGR1 in primary GBM cell cultures *in vitro* inhibits cell growth^35^, thereby suggesting EGR1 as a tumour suppressor in gliomas. Its anticancer effect is likely mediated through regulation of and interaction with key tumour suppressors such as PTEN and P53^36–38^.

When looking at EGR3 expression and patient survival, we found that high EGR3 expression levels were associated with poor patient prognosis. We found an increase in EGR3 protein expression in migrating tumour cells in the periphery, suggesting that EGR3 plays a role in tumour cell migration, thereby indirectly causing accelerated tumour progression, which leads to poor patient outcome. Supporting this hypothesis, a decline of EGR3 expression has been shown to decrease motility of non-small cell lung cancer cells^39^, while EGR3 has been associated with migration and invasion in hepatocellular^40,41^ and breast carcinomas^21^.

We did not find any association between cytoplasmic EGR3 localization and patient survival. However, the observed cytoplasmic EGR3 expression may have several functional implications; first it could indicate that tumour cells with this expression pattern have a high synthesis of EGR3 protein within the endoplasmatic reticulum, where it accumulates prior to nuclear translocation. Secondly, it has been shown that EGR3 protein accumulates around the microtubule organizing centers in dividing cells and is associated with microtubule formation^42^, thereby suggesting roles in cell division and cytoskeleton organisation. The exact functional role of cytoplasmic EGR3 remains elusive, and should be further investigated with functional assays.

High nuclear levels of EGR3 remained significantly associated with poor patient survival in MGMT-methylated patients after adjustment for confounders, as did the combination of EGR1 high/EGR3 high compared to EGR1 high/EGR3 low. However, since the vast majority of patients in the EGR1 high group had methylated MGMT promoters, the composition of the EGR1 high/EGR3 high group closely resembled the EGR3 high group sub-stratified from MGMT-methylated patients, and hence the combinations of EGR1 and EGR3 expression levels did not augment the prognostic value of either marker.

MGMT-methylation is predictive of positive response to temozolomide chemotherapy, and the poor prognosis of patients with high EGR3 expression in this group does raise the question whether EGR3 is implicated in resistance to temozolomide chemotherapy. EGR3 gene expression has previously been shown to increase in breast-cancer-associated fibroblasts after treatment with taxotere^43^, and furthermore it has been shown that tissue samples from patients with recurrent breast cancer had elevated levels of EGR3 compared to the matched primary tumours^44^. In gastric and colon cancer cell lines, it has been shown that EGR3 has binding sites in several genes related to 5-fluorouracil resistance^45^. This supports the hypothesis that EGR3 may have a protective function against chemotherapy, and although no direct association between EGR3 and temozolomide resistance has been described previously, it might be a relevant mechanism that could partly explain our findings.

Overall, our findings suggest that high EGR3 protein expression was associated with a poor prognosis in GBM patients with a methylated MGMT-promoter, while EGR1 expression was not associated with prognosis after adjustment for clinically relevant confounders. To our knowledge, EGR3 has not previously been investigated in gliomas, and our findings raise new questions about the role of EGR3 in GBM biology; the results indicate that EGR3 may be implicated in GBM cell migration and possibly also chemoresistance – two main features associated with treatment resistance and poor patient prognosis. Future research should aim to investigate and validate a potential role of EGR1 in the context of MGMT-methylation and the biological implication and prognostic value of EGR3 in GBMs.

## Supplementary information

Supplementary information.

## References

[1] Darefsky AS, King JT, Dubrow R (2012). Adult glioblastoma multiforme survival in the temozolomide era: a population-based analysis of Surveillance, Epidemiology, and End Results registries. Cancer..

[2] Stupp R (2009). Effects of radiotherapy with concomitant and adjuvant temozolomide versus radiotherapy alone on survival in glioblastoma in a randomised phase III study: 5-year analysis of the EORTC-NCIC trial. The Lancet Oncology..

[3] Omuro A, DeAngelis LM (2013). Glioblastoma and other malignant gliomas: a clinical review. Jama..

[4] Binabaj MM (2018). The prognostic value of MGMT promoter methylation in glioblastoma: A meta-analysis of clinical trials. Journal of cellular physiology..

[5] Brandner S, von Deimling AD (2015). prognostic and predictive relevance of molecular markers in gliomas. Neuropathology and applied neurobiology..

[6] Munthe S. *et al*. Migrating glioma cells express stem cell markers and give rise to new tumors upon xenografting. *Journal of neuro-oncology*. 2016. 10.1007/s11060-016-2221-y PMC5069331 27510953

[7] Pagel JI, Deindl E (2011). Early growth response 1–a transcription factor in the crossfire of signal transduction cascades. Indian journal of biochemistry & biophysics..

[8] DeLigio JT, Zorio DA (2009). Early growth response 1 (EGR1): a gene with as many names as biological functions. Cancer biology & therapy..

[9] Taefehshokr S, Key YA, Khakpour M, Dadebighlu P, Oveisi A (2017). Early growth response 2 and Egr3 are unique regulators in immune system. Central-European journal of immunology..

[10] Morita K (2016). Emerging roles of Egr2 and Egr3 in the control of systemic autoimmunity. Rheumatology (Oxford, England)..

[11] Parkinson RM, Collins SL, Horton MR, Powell JD (2014). Egr3 induces a Th17 response by promoting the development of gammadelta T cells. PloS one..

[12] Liu D, Evans I, Britton G, Zachary I (2008). The zinc-finger transcription factor, early growth response 3, mediates VEGF-induced angiogenesis. Oncogene..

[13] Suehiro J, Hamakubo T, Kodama T, Aird WC, Minami T (2010). Vascular endothelial growth factor activation of endothelial cells is mediated by early growth response-3. Blood..

[14] Sakakini N (2016). A Positive Feed-forward Loop Associating EGR1 and PDGFA Promotes Proliferation and Self-renewal in Glioblastoma Stem Cells. The Journal of biological chemistry..

[15] Riddick G (2017). A Core Regulatory Circuit in Glioblastoma Stem Cells Links MAPK Activation to a Transcriptional Program of Neural Stem Cell Identity. Scientific reports..

[16] Myung E (2013). Expression of early growth response-1 in human gastric cancer and its relationship with tumor cell behaviors and prognosis. Pathology, research and practice..

[17] Myung DS (2014). Expression of early growth response-1 in colorectal cancer and its relation to tumor cell proliferation and apoptosis. Oncology reports..

[18] Kumar SS (2017). High early growth response 1 (EGR1) expression correlates with resistance to anti-EGFR treatment *in vitro* and with poorer outcome in metastatic colorectal cancer patients treated with cetuximab. Clinical & translational oncology: official publication of the Federation of Spanish Oncology Societies and of the National Cancer Institute of Mexico..

[19] Kataoka F (2012). EGRI and FOSB gene expressions in cancer stroma are independent prognostic indicators for epithelial ovarian cancer receiving standard therapy. Genes, chromosomes & cancer..

[20] Liao F (2013). Decreased EGR3 expression is related to poor prognosis in patients with gastric cancer. Journal of molecular histology..

[21] Suzuki T (2007). Early growth responsive gene 3 in human breast carcinoma: a regulator of estrogen-meditated invasion and a potent prognostic factor. Endocrine-related cancer..

[22] Louis DN (2016). The 2016 World Health Organization Classification of Tumors of the Central Nervous System: a summary. Acta neuropathologica..

[23] Puchalski RB (2018). An anatomic transcriptional atlas of human glioblastoma. Science (New York, NY)..

[24] Bowman RL, Wang Q, Carro A, Verhaak RG, Squatrito M (2017). GlioVis data portal for visualization and analysis of brain tumor expression datasets. Neuro-oncology..

[25] Verhaak RG (2010). Integrated genomic analysis identifies clinically relevant subtypes of glioblastoma characterized by abnormalities in PDGFRA, IDH1, EGFR, and NF1. Cancer cell..

[26] Mittelbronn M (2009). EGR-1 is regulated by N-methyl-D-aspartate-receptor stimulation and associated with patient survival in human high grade astrocytomas. Brain pathology (Zurich, Switzerland)..

[27] Lim CP, Jain N, Cao X (1998). Stress-induced immediate-early gene, egr-1, involves activation of p38/JNK1. Oncogene..

[28] Yan SF (1999). Hypoxia-associated induction of early growth response-1 gene expression. The Journal of biological chemistry..

[29] Chen Y (2013). MGMT promoter methylation and glioblastoma prognosis: a systematic review and meta-analysis. Archives of medical research..

[30] Maifrede S (2017). Loss of Egr1, a human del5q gene, accelerates BCR-ABL driven chronic myelogenous leukemia. Oncotarget..

[31] Mohamad T, Kazim N, Adhikari A, Davie JK (2018). EGR1 interacts with TBX2 and functions as a tumor suppressor in rhabdomyosarcoma. Oncotarget..

[32] Choi EJ, Yoo NJ, Kim MS, An CH, Lee SH (2016). Putative Tumor Suppressor Genes EGR1 and BRSK1 Are Mutated in Gastric and Colorectal Cancers. Oncology..

[33] Baron V (2003). Inhibition of Egr-1 expression reverses transformation of prostate cancer cells *in vitro* and *in vivo*. Oncogene..

[34] Baron V, Duss S, Rhim J, Mercola D (2003). Antisense to the early growth response-1 gene (Egr-1) inhibits prostate tumor development in TRAMP mice. Annals of the New York Academy of Sciences..

[35] Calogero A (2004). Inhibition of cell growth by EGR-1 in human primary cultures from malignant glioma. Cancer cell international..

[36] Zwang Y (2011). Two phases of mitogenic signaling unveil roles for p53 and EGR1 in elimination of inconsistent growth signals. Molecular cell..

[37] Baron V, Adamson ED, Calogero A, Ragona G, Mercola D (2006). The transcription factor Egr1 is a direct regulator of multiple tumor suppressors including TGFbeta1, PTEN, p53, and fibronectin. Cancer gene therapy..

[38] Kim J (2014). EGR1-dependent PTEN upregulation by 2-benzoyloxycinnamaldehyde attenuates cell invasion and EMT in colon cancer. Cancer letters..

[39] Chien MH (2017). KSRP suppresses cell invasion and metastasis through miR-23a-mediated EGR3 mRNA degradation in non-small cell lung cancer. Biochimica et biophysica acta Gene regulatory mechanisms..

[40] Wang ZD (2017). Involvement of microRNA-718, a new regulator of EGR3, in regulation of malignant phenotype of HCC cells. Journal of Zhejiang University Science B..

[41] Li W (2015). Regulation of tumorigenesis and metastasis of hepatocellular carcinoma tumor endothelial cells by microRNA-3178 and underlying mechanism. Biochemical and biophysical research communications..

[42] Shin H, Kwon S, Song H, Lim HJ (2014). The transcription factor Egr3 is a putative component of the microtubule organizing center in mouse oocytes. PloS one..

[43] Li Y, Rong G, Kang H (2017). Taxotere-induced elevated expression of IL8 in carcinoma-associated fibroblasts of breast invasive ductal cancer. Oncology letters..

[44] Vareslija D (2016). Adaptation to AI Therapy in Breast Cancer Can Induce Dynamic Alterations in ER Activity Resulting in Estrogen-Independent Metastatic Tumors. Clinical cancer research: an official journal of the American Association for Cancer Research..

[45] Szoke D (2007). Identification of consensus genes and key regulatory elements in 5-fluorouracil resistance in gastric and colon cancer. Onkologie..

