# Supplementary material for: Expression and prognostic value of the transcription factors EGR1 and EGR3 in gliomas
Source: Sci Rep. 2020 Jun 9;10:9285. doi: 10.1038/s41598-020-66236-x (PMC7283475; doi:10.1038/s41598-020-66236-x)
Supplement: Supplementary file 1 — Supplementary information. [file 41598_2020_66236_MOESM1_ESM.pdf]

Expression and prognostic value of the transcription factors EGR1 and EGR3 in gliomas

Arnon Møldrup Knudsen, Ida Eilertsen, Susanne Kielland, Mikkel Warming Pedersen, Mia Dahl Sørensen, Rikke Hedegaard Dahlrot, Henning Bünsow Boldt, Sune Munthe, Frantz Rom Poulsen, Bjarne Winther Kristensen.

Supplemental figure 1

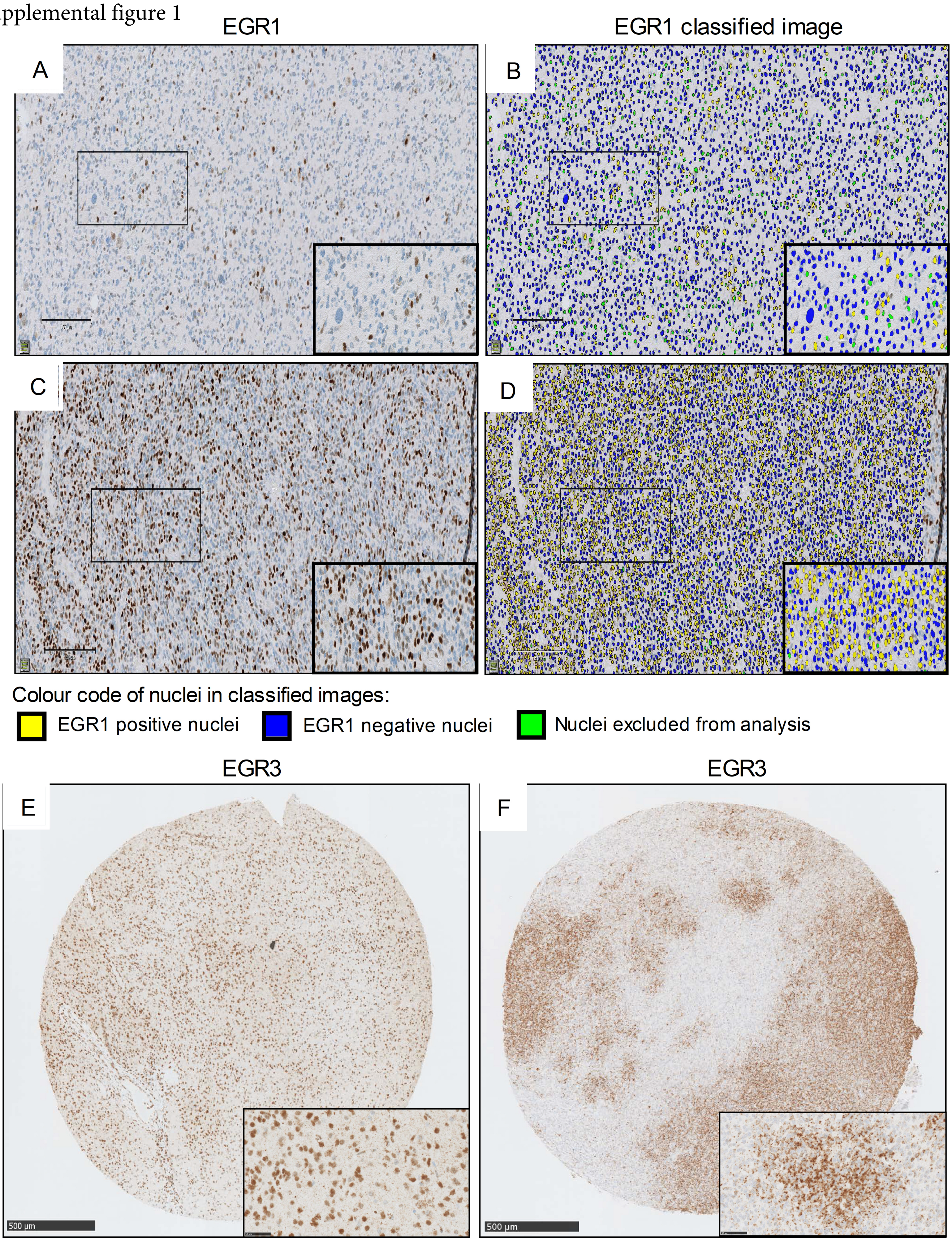

G

Category	Score criterion	Score	Core E	Core F
Fraction of positive nuclei (%)	0% - <10% positive nuclei<br>10% - <40% positive nuclei<br>40% - <70% positive nuclei<br>>=70% positive nuclei	0<br>1<br>2<br>3	3	1
Mean staining intensity of positive nuclei	No staining<br>Weak staining<br>Moderate staining<br>Strong staining	0<br>1<br>2<br>3	3	3
Fraction of cytoplasmic staining (% of total core area)	0 % - <5% tumor area<br>5% - <15% tumor area<br>15% - <25% tumor area<br>>=25% tumor area	0<br>1<br>2<br>3	0	3
Total score		0-9	6	7

Supplemental figure 2

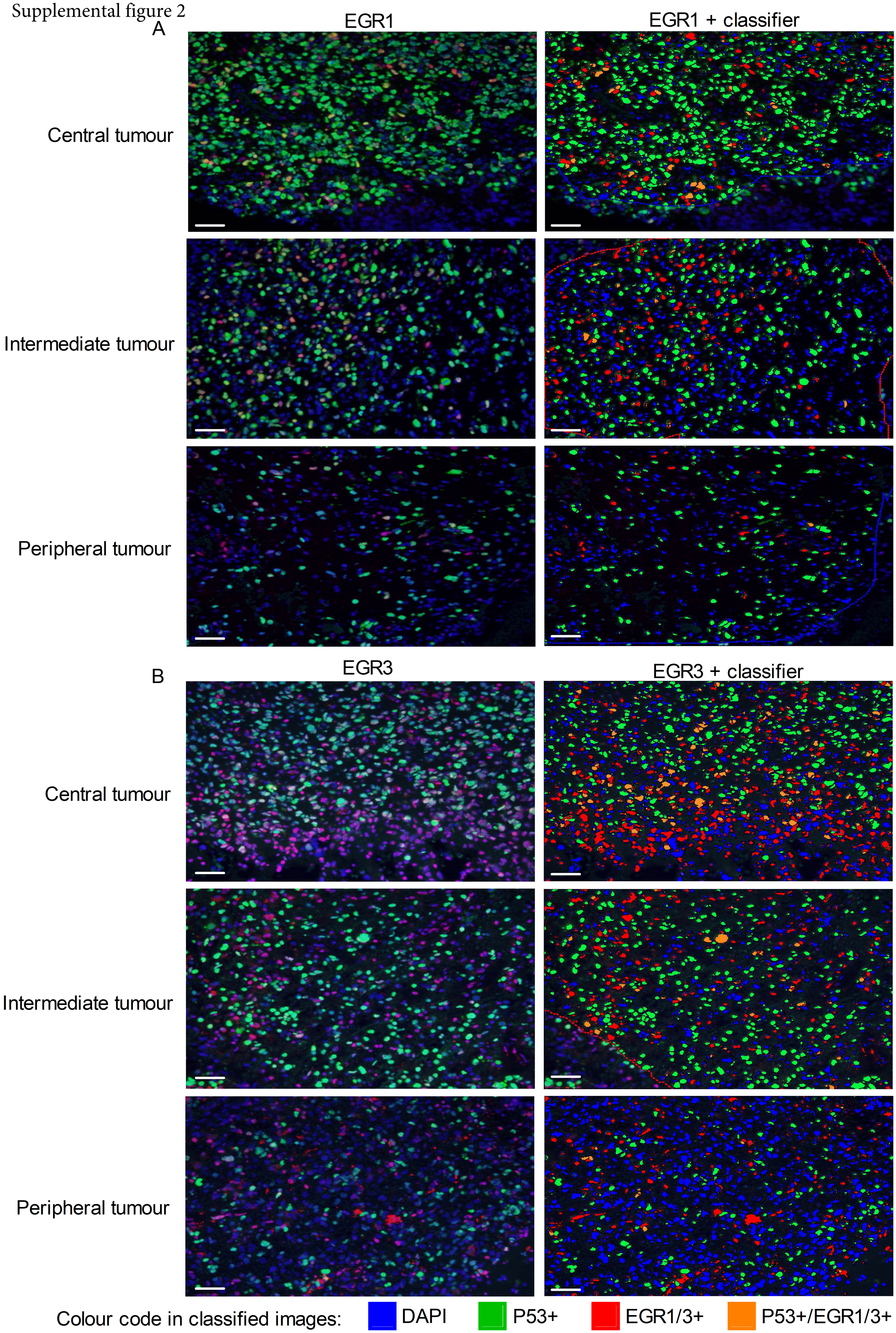

Supplemental figure 3

A

Interaction between age and MGMT-methylation status.

Variable	No. of patients	HR (95% CI)	P-value
Age	190	1.02 (1.01-1.04)	**0.004**
MGMT-methylation status			
Unmethylated	71	1.00	
Methylated	94	0.54 (0.38-0.77)	**0.001**
Age*MGMT Methylated		1.04 (1.01-1.08)	**0.013**
Post-surgical treatment			
No	18	1.00	
Yes	172	0.57 (0.31-1.05)	0.073
Performance status			
0-1	135	1.00	
2-4	54	3.20 (2.12-4.85)	**<0.001**
Gender			
Female	82	1.00	
Male	108	0.81 (0.58-1.14)	0.23

Age (years)	MGMT methylation status		Total
	Un-methylated	Methylated	
0-40	3	4	7
41-50	4	8	12
51-60	14	24	38
61-70	23	28	51
71-80	23	25	48
81+	4	5	9
Total	71	94	165

B

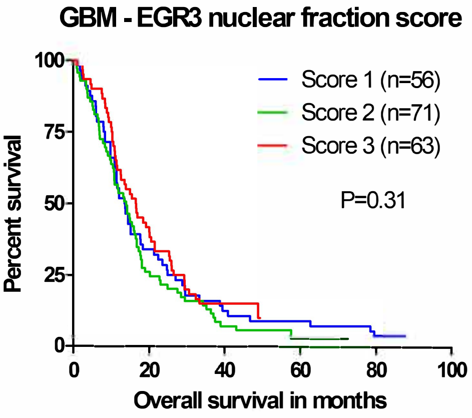

C

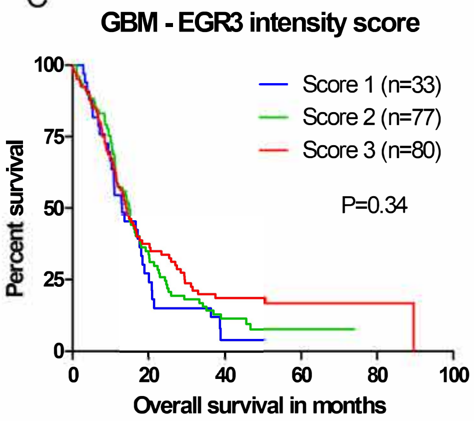

D

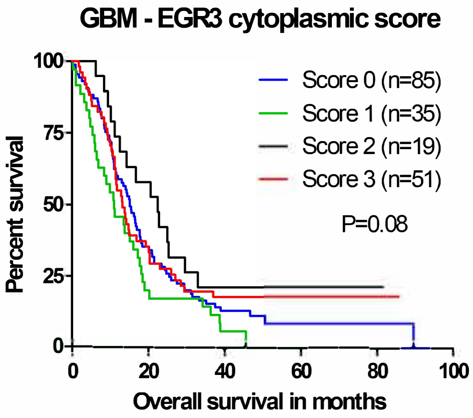

E

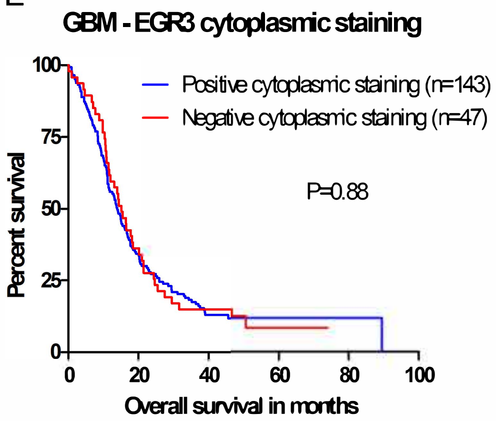

F

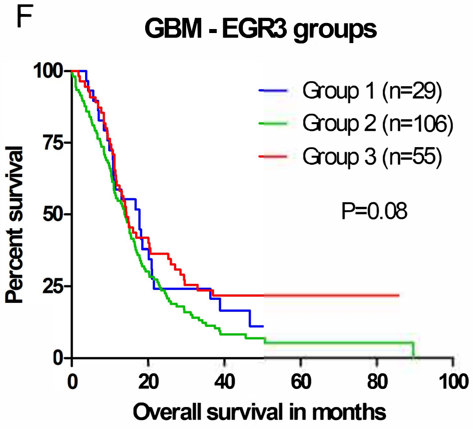

G

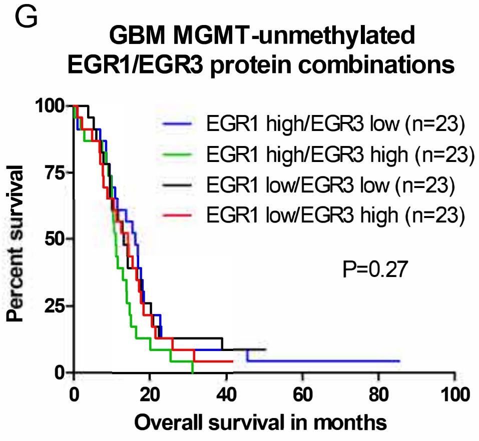

Supplemental figure legends

Supporting figure 1. Digital classification of EGR1 immunostainings and semi-quantitative scoring system for EGR3 stainings. **A-B)** GBM with a low EGR1-positive fraction (10%). **C-D)** GBM with high cell density and closely adjacent EGR1-positive cells. **E)** Tumour core with a specific nuclear EGR3 staining pattern and a high fraction of EGR3 positive nuclei. **F)** Tumour core with diffuse areas of cytoplasmic staining, and low nuclear staining fraction. **G**) Schematic overview of scoring categories and criterions for the semi-quantitative scoring of EGR3 stainings. Scale bars in A-D=150 µm and E-F=500 µm with insets = 50 µm. Magnification of images A-D=X10 with inset magnification=X40; E-F=X4 with insets =X40.

Supporting figure 2. Representative examples of the software-based cell classifier quantifying migrating tumour cells. A) EGR1 immunofluorescence stainings from the three different tumour regions depicted in the left column with the classifier applied on adjacent images in the right column. B) EGR3 immunofluorescence stainings shown as described above. Scale bar = 50 µm.

Supporting figure 3. Cox-regression interaction term and non-significant Kaplan-Meier survival data.
A) While performing interaction tests for Cox-regressions, a significant interaction was found between patient age and MGMT-methylation status, with older patients being more likely to have a methylated MGMT-promoter. **B-D)** Division of GBM patients based on their EGR3 nuclear fraction scores, intensity scores or cytoplasmic scores did not show any differences in survival between the groups. **E)** Presence or absence of EGR3 cytoplasmic staining did not have any impact on patient survival. **F)** The survival of patients in assigned groups based on total points achieved in the EGR3 scoring system did not show any significant differences across the groups. **G)** Different EGR1 and EGR3 protein combinations in MGMT-promoter un-methylated GBMs did not differ significantly when investigating patient survival.
